# Clinical Applications of Liquid Biopsy in Gastric Cancer

**DOI:** 10.3389/fmed.2021.749250

**Published:** 2021-09-28

**Authors:** Mihaela Chivu-Economescu, Laura Necula, Lilia Matei, Denisa Dragu, Coralia Bleotu, Carmen C. Diaconu

**Affiliations:** ^1^Department of Cellular and Molecular Pathology, Stefan S. Nicolau Institute of Virology, Bucharest, Romania; ^2^Faculty of Medicine, Titu Maiorescu University, Bucharest, Romania

**Keywords:** liquid biopsy, gastric cancer, screening, prognosis, circulating tumor cells (CTCs), circulating tumor DNA (ctDNA), circulating non-coding RNAs (ncRNA), exosomes

## Abstract

Liquid biopsy represents an exciting new area in the field of cancer diagnosis and management, offering a less invasive and more convenient approach to obtain a time-point image of the tumor burden and its genomic profile. Samples collected from several body fluids, mostly blood, can be used to gain access to circulating tumor cells and DNA, non-coding RNAs, microRNAs, and exosomes, at any moment, offering a dynamic picture of the tumor. For patients with GC, the use of blood-based biopsies may be particularly beneficial since tissue biopsies are difficult to obtain and cause real distress to the patient. With advantages such as repeatability and minimal invasion, it is no wonder that the field of liquid biopsy has received tremendous attention. However, the abundance of studies, involving a wide range of assays with different principles, prevented for the moment the reproducibility of the results and therefore the translation into the clinic of liquid biopsy. In this review, we present the latest technical development and data on circulating biomarkers available through liquid biopsy in gastric cancer with an emphasis on their clinical utility in areas such as cancer screening, prognostic stratification, and therapeutic management.

## Introduction

Gastric cancer (GC) is the fifth most common cancer worldwide, with 1.089.103 new cases being registered in 2020 (5.6%), after female breast cancer (11.7%), lung (11.4%), colorectal (10.0 %), and prostate (7.3%). Compared with other cancers, morbidity rates are quite high. With 768.793 (7.7%) deaths each year, GC represents the fourth cause of cancer-related death in the world (1). To overcome this burden, significant efforts are being made by clinicians and researchers around the world.

To date, several serum biomarkers have been identified and widely used for GC diagnosis and prognosis, including carcinoembryonic antigen (CEA), cancer antigen 19-9 (CA19-9), cancer antigen 125 (CA125), and cancer antigen 72-4 (CA72-4). However, those biomarkers have low sensitivity (<40%), and their specificities are modest. Only when used as a triple marker, CEA, CA19-9, and CA72.4, the sensitivity was 62.0% (2). Moreover, they may offer a certain value only in determining peritoneal metastasis, estimating poor prognosis and a higher risk for recurrence of GC rather than in setting up a diagnosis (3). Preoperative levels of CEA, CA19-9, and CA125 were analyzed on 768 patients with GC and found above the cut-off levels in only 15.4, 8.7, and 5.7% of all cases, respectively (4). In a comprehensive meta-analysis of literature published on GC serum markers, the Japanese Gastric Cancer Association concluded that they are not useful for early cancer diagnosis, but they are useful for detecting recurrence and distant metastasis, predicting patient survival, and monitoring after surgery (5).

In this regard, liquid biopsy emerged as a promising tool for early detection, treatment selection, and real-time progression information. In the last decade, it became clear that closer monitoring of disease progression during second line and salvage chemotherapy proved to be extremely important for overall survival. In that aspect, novel technologies, like a liquid biopsy and new biomarkers offer guidance on the proper timing for therapy and impact assessment. Another major advantage of liquid biopsies consists in offering a rapid and precise, less invasive and more convenient approach to obtain cancer information. It may be particularly beneficial since biopsies are difficult to obtain and cause real distress to the patient.

**Figure 1:**
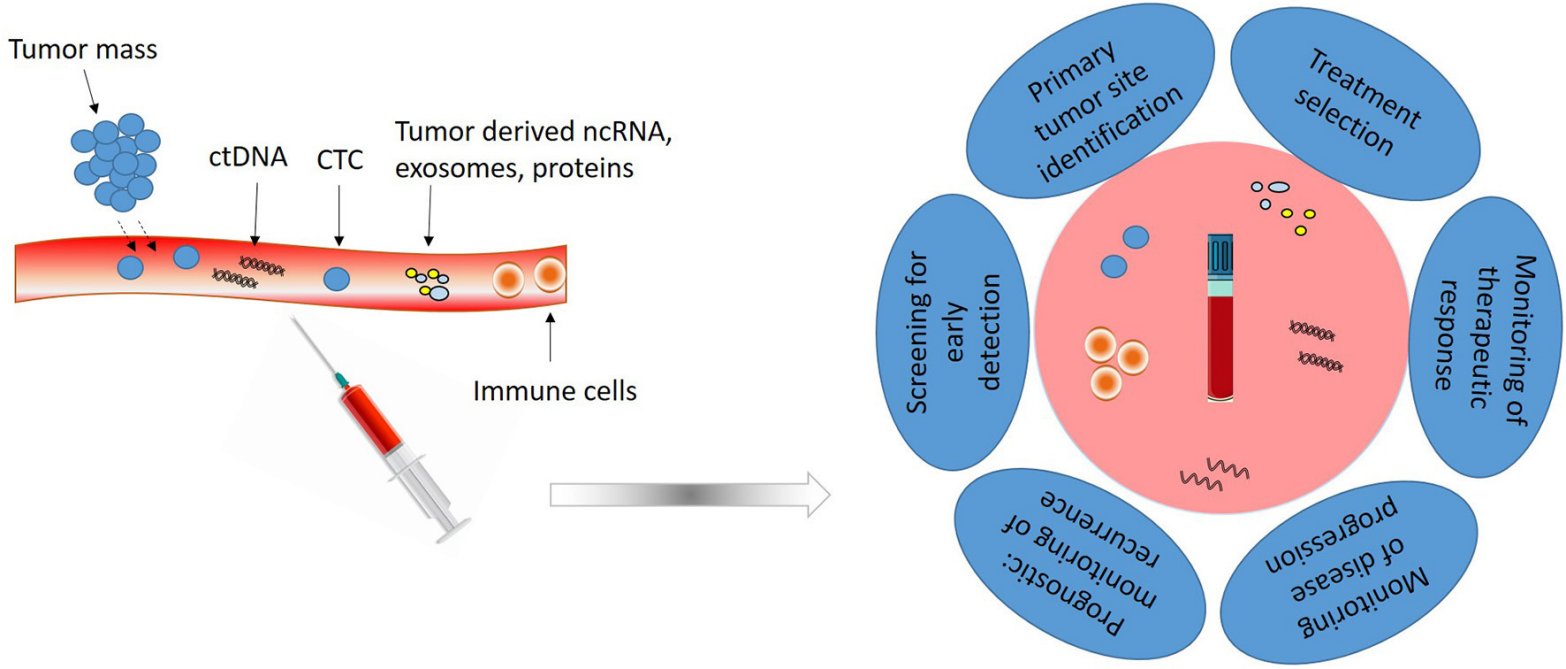
Liquid biopsy composition and utility. Liquid biopsy obtained from peripheral blood is composed of circulating tumor cells (CTCs), circulating tumor DNA (ctDNA), non-coding RNAs (ncRNAs) and different tumor derived proteins and vesicles. Analysis of these elements has clinical utility for early detection, treatment selection, real-time progression information and prognosis.

Samples can be collected from several biological fluids such as blood, saliva, breast milk, cerebrospinal fluid, urine, gastric juice, semen, etc., but the most used and characterized type is blood (Figure 1).

Nowadays, liquid biopsy cover for the investigation of circulating tumor cells (CTCs), circulating tumor DNA (ctDNA), circulating non-coding RNAs (ncRNAs), microRNAs, and circulating exosomes, in the blood of patients with cancer. It can assess more accurately the tumor dynamics and heterogeneity, monitor treatment responses and potential emergence of drug resistance, determine the minimal residual disease and indicate a personalized cancer management strategy. Liquid biopsies provide access to CTCs, DNA, and ncRNA, at any moment, with minimal discomfort for the patients, offering a dynamic picture of the tumor. However, the technology to obtain enough material for monitoring disease progression and drug resistance is labor-intensive, must have high sensitivity and specificity, and should permit multiplex determination of panels of relevant markers.

As today, the FDA has approved several technologies for monitoring cancer patients using liquid biopsy: CELLSEARCH CTC test for quantification of circulating tumor cells from Veridex, Guardant360 CDx and FoundationOne Liquid CDx, both using circulating cell-free DNA and next-generation sequencing (NGS) technology to provide tumor mutation profiling. These platforms have received clearance for malignancies such as metastatic lung, breast, prostate, or colorectal cancer. For GC clinical management, they have not yet been introduced in daily practice, although a great benefit for patients and clinicians could be anticipated, by overcoming limitations of traditional tissue biopsies.

## Circulating Tumor Cells (CTCs)

CTCs are cells derived from the primary tumor that infiltrate into the bloodstream, and from there on to other body fluids. CTCs are extremely rare events, with about one to 10 cells in 10 ml of peripheral blood (6). Since they represent the phenotypic and genotypic image of the primary tumor, identification and analysis of CTCs emerged as a promising assay to screen for early carcinogenesis, and to monitor cancer progression and treatment effectiveness in a real-time manner. CTCs are currently approved by the FDA as a prognostic biomarker and used in monitoring cancer evolution in patients with breast, prostate, and colorectal cancer (7). In GC, CTCs are very rare and difficult to identify, the best method of detection and specific markers remaining controversial.

Currently used methods for CTC isolation include a primary step of enrichment consisting on depletion of red and white blood cells known as negative selection, and a positive selection based on their biological and physical properties, with subsequent identification supported by immunological, molecular, and functional markers. There are several enrichment technologies known, such as Cell Search, Adna Test, CTC-chip, MACS, or MagSweeper, all of them using epithelial cell adhesion molecule (EpCAM) as a surface marker for enrichment (8). The main issue is that during the metastasis process, involving migration, intravasation, circulation into the bloodstream, and extravasation with new organ colonization, CTCs undergo epithelial to mesenchymal transition (EMT) and lose their epithelial markers becoming more difficult to identify. However, with EMT comes the overexpression of extracellular matrix (ECM) proteins such as vimentin, fibronectin, twist, ZEB1, ZEB2, snail, slug, and N-cadherin (9, 10). These could help more accurately identify CTCs if they are included in the existing enrichment technologies.

Other methodologies for the enrichment of CTCs are based on physical properties: size (isolation by size of epithelial tumor cells ISET) or density (OncoQuick, RosetteSep), but these can result in losses in the number of isolated CTCs.

After enrichment, follows the CTC identification step which is performed by immunocytological methods (detection of other specific surface antigens like cytokeratins (CK), 8, 18, 19, 20, CD44), molecular (amplification by qRT-PCR of specific CTC markers like surviving, CKs, VEGFR), or functional [epithelial immunospot EPISPOT that detects protein secretion (e.g., CK19)]. Of these methods, the one that has the highest specificity in identifying CTC is molecular testing, with the limitation related to the fact that cell viability is lost.

To date, CellSearch System (Veridex) was approved by FDA for clinical use in breast, colorectal, and prostate cancers (11–13). The system immunomagnetically enriches CTCs expressing EpCAM from 7.5 mL of blood using ferrofluids beads coated with anti-EpCAM antibodies and then selects fluorescently cells based on DAPI, CD45-APC, and 8, 18 and 19 CK-PE. Other system for positive selection of CTC is cytokeratin (CK)-dependent immunomagnetic separation system from Miltenyi based on anti-pan CK antibody that recognizes CK 7, 8, 18, and 19.

## Clinical Utility of CTCs

Several studies have shown that CTCs have significant values in the diagnosis, prognosis, and treatment management of GC at various stages (Table 1).

**Table 1:** Clinical utility of CTCs in GC.

**Study**	**Separation method**	**Cut-off number**	**Clinical utility**
Kang et al. (14)	“FAST disc” centrifugal microfluidic system	≥2/7.5 ml blood	Early diagnosis Assay sensitivity/specificity: 85.3/90.3%.
Liu et al. (15)	CELLection Epithelial Enrich kit	>2/5 ml blood	Treatment monitoring and prognosis based on CTC number evolution after chemotherapy. CTC positive rate −83.05%
Pernot et al. (16)	CellSearch	≥2/7.5 ml blood	Treatment monitoring and prognosis Assay sensitivity/specificity: 69/68%.
Chen et al. (17)	ISET device combining IF and Wright's staining	>1/5 ml blood	Progression CTC positive rate was associated with invasion, metastasis, and TNM stage. Assay sensitivity/specificity: 72.65/52.8%.
Yang et al. (18)	Microfluidic CTC-Δchip based on cells size	>3/2 ml blood	Diagnosis 7.30 ± 7.29 CTCs were detected from 2 mL peripheral blood with a positive rate of 75% (30/40)
Yue C et al. (19)	Pep@MNPs isolated system and characterized as CK19+ DAPI+ CD45-	>2/4 ml blood	Therapeutic management and monitoring of PD-L1 monoclonal antibody treatment. Detected up to 5 CTCs from 4 mL peripheral blood.
Li et al. (20)	Metafer-iFISH Cytelligen system	≥2 cHER2^+^ CTCs/6 ml blood	Treatment monitoring. Acquisition of HER2 phenotype on CTCs correlated with resistance to trastuzumab.
Cheng et al. (21)	CanPatrol CTC enrichment technique	≥2 CTC-PD-L1+/5 ml blood	Therapeutic management and monitoring of PD-L1 monoclonal antibody treatment.
Ning et al. (22)	CellSearch, CTC-Biopsy	≥2 CTC/7.5 ml blood	Prognosis. >3 CTC correlated with reduced progression-free survival and overall survival.

CTCs detection has been suggested to be a useful biomarker for diagnosis. In a study conducted by Kang HM el al. using a CTC level of ≥2 per 7.5 mL of blood, the authors successfully differentiate patients with GC from healthy controls. Sensitivity and specificity were 85.3 and 90.3%, respectively (14). In addition, CTCs were identified in more than 80% of early-stage (T1 or N0) cases, suggesting that CTCs could be an early diagnostic biomarker for GC with high sensitivity and specificity.

As bloodstream circulating cells detached from the primary tumor, CTCs are significantly involved in cancer metastasis and recurrence. As a result, it is not surprising that many studies have shown that the presence of CTC is associated with advanced stages, poor survival, and progression-free survival in GC (23). In a meta-analysis performed on 3,814 GC patients, Gao Y et al. concludes that the presence of CTCs identifies a group of patients with poor overall survival (OS) (CTCs: HR = 1.84, 95% CI 1.50–2.26, *p* < 0.001) (24).

In addition to diagnosis and prediction of prognosis, several studies reported that monitoring changes in the number of CTCs during treatment may be a predictive marker of treatment response. Li et al. (25) showed that high CTCs numbers (≥3) during treatment and post-therapy correlates with ineffective therapeutic response and reduced PFS and OS. In another study (26), on 100 patients with metastatic GC, was found that a CTCs number ≥5 during palliative chemotherapy is associated with limited response to the treatment and poor prognosis. Similar results were obtained in another study (27), in which quantification of CTCs in patients with advanced GC at 2 or 4 weeks after initiation of the treatment (S-1-based, with or without cisplatin, or base on paclitaxel) demonstrated that patients with ≥4 CTCs at 4 weeks had 3.5 months inferior median OS than the patient with <4 CTCs.

Besides CTC counting, molecular and phenotypic characterization of CTCs may provide additional information on the mechanism of therapy resistance and recurrence risk in patients with GC. This date is sustained by Szczepanik et al. (28), who demonstrated that only a subpopulation of CTCs expressing cytokeratins (8, 18, and 19) and CD44 marker (CK+CD44+) are significantly associated with distant metastases and with reduced survival.

New biomarkers such as the HER2 and PD-L1 positivity or amplification on CTCs are nowadays considered useful in predicting a positive response to trastuzumab and checkpoint therapy. For instance, HER2-positive CTCs were detected in nonmetastatic gastric adenocarcinoma patients with disease progression but HER2-negative primary tumors. Mishima et al. (29) demonstrated that a re-evaluation of HER-2 status on CTCs in advanced GC patients (*n* = 15) whose primary tumors were HER2-, but HER2 positive CTCs, may have clinical implications, allowing a better selection of patients for trastuzumab therapies (29, 30).

Moreover, two studies based on immune-checkpoints detection on CTC PD-L1 demonstrated that the abundance of PD-L1-positive CTCs compared with baseline might be used to screen patients with GC that will most likely benefit from PD-1/PD-L1 blockade therapies (19, 21). Moreover, dynamic changes of PD-L1 positive CTC count might be used to monitor treatment response as a non-invasive strategy.

## Circulating Tumor DNA (ctDNA)

ctDNA originates as a result of different physiological events like apoptosis, necroptosis, tumor secretion, and micro-metastasis, and can be detected in different body fluids like blood serum or plasma, synovial fluid, cerebrospinal fluid, etc. In cancer patients, circulating tumor DNA (ctDNA) can have up to four- or five-times greater levels than circulating free DNA (cfDNA) in healthy controls and harbor tumor-associated molecular alterations (31). It offers the possibility to explore alterations in tumor DNA at the genetic and epigenetic level through assays such as copy number variations (CNVs), gene integrity, gene mutations, DNA methylation, and therefore proved to be essential to study tumor genomics distinguishing molecular subtypes, screening of EBV-associated cancers, treatment selection based on detected mutations in ctDNA, and to follow tumor progression and clonal evolution of tumors, at regular bases without difficulties in obtaining serial biopsies (32, 33).

Nevertheless, there are several limitations in performing ctDNA analyses: the detection and quantification of ctDNA are limited by its instability and kinetics, and also ctDNA concentration is highly variable, being very low especially in early stage cancers, and requires highly sensitive detection methods. The detection of ctDNA is facilitated if the primary tumor genetic abnormalities are already known.

Currently developed methods for ctDNA-based assays are varying widely between detection of a single-point mutation and the analysis of the entire genome; techniques such as PCR or PCR-based assays (qPCR, ddPCR, ARMS, MS-PCR, etc.) or targeted sequencing are used. Nowadays, novel high throughput sequencing techniques such as NGS or WES can identify mutations in multiple genetic regions (34, 35). Thus, two platforms based on NGS have already been approved by the FDA for the analysis of genomic profile in cancer patients, by liquid biopsy. Guardant360 CDx received FDA approval for clinical use in August 2020. The analysis facilitates the detection of single nucleotide variants (SNVs), insertions and deletions (indels) in 55 genes, copy number amplifications (CNAs) in two genes, and fusions in four genes. The panel is indicated for the clinical management of patients with non-small cell long cancer (NSCL) (36, 37). Also in August 2020, FDA has approved the FoundationOne Liquid CDx, which uses NGS technology optimized for cfDNA to investigate >300 genes. It detects major types of genomic alterations, microsatellite instability, blood tumor mutational burden, and tumor fraction values. FoundationOne Liquid CDx system was validated on several types of solid cancers, including mostly NSCLC, prostate, ovarian and breast cancer samples. The assay performance studies were conducted on cfDNA isolated from plasma of cancer patients representing 37 cancer types, including gastric cancer (38).

## Clinical Utility of ctDNA

The analysis of ctDNA can be used in the detection of different genetic and epigenetic alterations, microsatellite instability, deletion, amplification, chromosome translocation, and loss of heterozygosity. The results of some recent studies in the GC field being reported in Table 2.

**Table 2:** ctDNA as biomarker in GC.

**Study**	**Detection method**	**Marker/Values**	**Clinical utility**
Kim et al. (39)	NGS	ctDNA at 6 weeks post-treatment	Treatment monitoring and prognosis. Decreased ctDNA was associated with improved outcomes and progression-free survival in metastatic GC EBV+ or MSI-H treated with pembrolizumab.
Chen et al. (40)	WGS	chromosomal instability assessed by copy number instability (CNI) score of ctDNA	Predict and monitor therapeutic response in GC.
Kim et al. (41)	WGS	ctDNA personalized cancer-specific rearrangements	Surveillance for recurrent disease after curative surgical resection. The median lead time was 4.05 months.
Lan et al. (42)	NGS	ctDNA	Disease monitoring. Presence of ctDNA correlated with metastasis lymph node number and with lactate dehydrogenase level.
Wang et al. (43)	NGS	ctDNA mTBI sensitivity 94%	Prognosis. Identified disease progression before imaging results (median time 18 weeks)
Grenda et al. (44)	qPCR	*HER2* CNV sensitivity 43%/specificity 100%	Diagnostic. Use HER2 copy number detected in ctDNA to distinguish patients with HER2 positive GC from healthy individuals.
Yan et al. (45)	methylation-specific PCR	*SFRP2* hypermethylation	Prognosis prediction and dynamic monitoring among GC.
Yang et al. (46)	NGS	ctDNA At 90 days: Sensitivity 100%; Specificity 84 % At 900 days: Sensitivity 39%; Specificity 100%	Disease monitoring. Identified patients at high risk for recurrence after definitive therapy (median lead time−6 months). Prognosis. ctDNA was associated with worse disease-free and overall survival.
Ko et al. (47)	HELP (HpaII tiny fragment Enrichment by Ligation-mediated PCR)	long-and short-fragment LINE-1 in cfDNA	Prognosis. Pre-surgical low methylation levels of LINE-1 were a negative prognostic factor. Disease monitoring. Post-surgical high concentrations of long-fragment LINE-1 indicated MRD and a high risk of recurrence.
Li et al. (48)	NGS	*TP53* mutations *MET* amplification	Disease progression based on detection of TP53 mutation and MET amplification.

Usually, ctDNA can be detected before therapy and disappears after complete surgical resection. Its presence after or its reappearance at follow-up indicates minimal residual disease (MRD), which is a cause of recurrence (41, 42, 46, 47, 49, 50).

The analysis of ctDNA can be used to guide treatment decisions and evaluate clinical response. Thus, Zhang M et al. evaluated the ctDNA genomic profile of Chinese advanced GC patients by NGS and determined genomic alterations (like del-alterations in TP53, LRP1B, MYC, ERBB2, and KRAS genes), blood tumor mutation burden, and blood microsatellite instability status that can advise the clinical decision in advanced GC (51). Analyzing the profile of ctDNA genetic alterations in a cohort of 46 patients with GC, Iqbal M et al.established that only a few genes are altered, in the top 11 being TP53, KRAS, PIK3CA, ARID1A, EGFR, APC, ERBB2/HER2, CDK6, MET, PTEN, and MYC, presenting single nucleotide variations, CNV, and indels (32).

Kim ST et al.used Guardant360 ctDNA NGS assay to assess tumor mutational load and to determine the microsatellite instability (MSI) status in pretreatment tissue aiming to identify patients with metastatic gastric cancer who are most likely to benefit from pembrolizumab treatment. In addition, patients were followed with a serial collection of plasma-derived ctDNA. The obtained results showed a decrease in ctDNA level at six weeks post-treatment, which predicted response to immune chechpoint therapy and was associated with improved outcomes and progression-free survival (39).

ctDNA can be used for HER2-targeted population screening, since there is a high concordance of HER2 amplification between ctDNA and tumor tissues (52, 53). Also, HER2 CNV detected in ctDNA could be used to monitor trastuzumab efficacy (52, 54, 55), as well as to predict innate trastuzumab resistance (54) and the developed one (43).

In the VIKTORY trial (NCT 02299648), high MET copy number in ctDNA correlated with response to savolitinib, an experimental small-molecule inhibitor of c-Met. The concordance rate between tumor and ctDNA for MET amplification was 89.5%, with 100% specificity and 83.3% sensitivity relative to tissue testing, which increased to 100% when patients without detectable ctDNA were excluded (56). Du J et al.also reported the potential of ctDNA profiling for treatment decision and prognosis in a case of a stage IV GC patient with high levels of MET amplification.

Periodic mutation profiling of ctDNA by NGS revealed the appearance of several genetic alterations including re-occurrence of MET amplification, multiple secondary MET mutations, a dramatic increase of FGFR2 gene relative copy number as well as mutations in other downstream and bypassing elements that were correlated with the patient's cancer progression, transient response, and resistance development to crizotinib treatment, a receptor tyrosine kinase inhibitor (57). Furthermore, the utility of ctDNA monitoring in resistance development during treatment and progression was reported by Frigault MM et al. who identified the mechanisms of acquired resistance to savolitinib in three patients with GC and MET-amplified tumors that showed a clinical response followed by cancer progression (58).

## Circulating Non-coding RNAs (ncRNAs)

The development of RNAseq techniques highlighted the circulating transcriptome, including coding and non-coding RNA, as an important source of potential diagnostic, prognostic, or predictive cancer biomarkers (59). Aberrant expression of circulating non-coding RNAs (ncRNAs), mostly microRNAs (miRNAs) and long non-coding RNAs (lncRNAs), has been reported in several cancers, including GC, where these molecules seem to act as tumor suppressors or oncogenes, mediating important intracellular processes. Therefore, many studies suggested that an increased/decreased of ncRNA expression in blood could guide the therapeutic approach being associated with disease grade, malignant progression, or treatment response (60).

ncRNAs are usually secreted in the blood, urine, or other body fluids as a result of cell necrosis, apoptosis, or due to an active secretion of the tumor cells and these molecules are stable enough to be manipulated since they are protected by exosomes and microvesicles. Therefore, analyzing the expression of circulating ncRNAs could bring important information about the expression profile of the primary tumor (61).

miRNAs could be considered promising GC biomarkers since many studies reported an aberrant expression of these molecules in gastric tissue in preneoplastic events, such as Helicobacter pylori infection, chronic gastritis, preneoplastic conditions (atrophic gastritis, intestinal metaplasia), and also in early and advanced cancer (62). Circulating lncRNAs expression was also correlated with diagnosis, prognosis, and therapy monitoring of GC patients (60, 63).

## Clinical Utility of Non-coding RNAs (ncRNAs)

A recent study showed that an increased expression of miR-1246 in serum could distinguish between GC patients with TNM stage I, healthy controls, and patients with benign diseases, highlighting the utility of this molecule as a biomarker for early diagnosis of GC. This elevated serum level of miR-1246 seems to be tumor-derived and it is packaged into exosomes (64). miR-212, a molecule that is epigenetically downregulated in GC showed a decreased expression in serum of GC patients compared with healthy donors, being negatively correlated with tumor stages (65). Low serum levels of miR-203 are associated with lymph node, peritoneal, and distant metastases in GC patients (66) while a high level of miR-21-5p could be detected in urine samples of GC patients and this level is reduced after the surgical removal of the tumors (67).

In Table 3 are presented several ncRNAs that are found to be differentially expressed in GC patients and are associated with prognostic and therapy monitoring. However, more studies are needed to validate a panel of ncRNA biomarkers for GC.

**Table 3:** Aberrant expression of circulating non-coding RNAs in GC patients.

**Study**	**circulating non-coding RNAs**	**Origin of sample**	**Clinical utility**	**Sensitivity/Specificity**
Wang et al. (68)	↑miR-106a-5p, miR-19b-3p	serum/exosomes	Prognostic/Progression. Discriminates between GC patients and healthy controls; expressed at higher levels in stages III and IV compared to I and II stages; related to GC lymphatic metastasis.	95/90%
Tang et al. (69)	↓circ-KIAA1244	plasma/exosomes	Prognostic/Progression. Decreased level of circ-KIAA1244 negatively correlated with TNM stage, lymphatic metastasis, and shorter overall survival of GC patients	77.42/68.00%
Zhao et al. (70)	↑lncRNA HOTTIP	serum/exosomes	Prognostic/Progression. Upregulated in GC, correlated with invasion depth, TNM stage, and poor overall survival.	69.8/85.0%
Shiotani et al. (71)	↑miR-106b, ↑miR-21	serum	Prognostic. Markers of increased risk for early GC after H. pylori eradication	69.0/69.4%
Chen et al. (72)	↑miRNA-22-3p	plasma	Prognostic. Predicts the malignant progression of precancerous gastric lesions to intestinal metaplasia and early adenocarcinoma	91.7/65.40%
Xian et al. (73)	↑ZNFX1-AS1, ↑HULC	plasma	Diagnostic. Increased in GC patients compared with gastrointestinal stromal tumor patients, gastritis/peptic ulcer patients and control group	84/68%, 58/80%
Lin et al. (74)	↑lncUEGC1	plasma/ exosomes	Diagnostic. Discriminates between early GC and healthy controls	88.24/83.33%
Chen et al. (75)	↑miRNA-196a	Plasma	Diagnostic. Higher in patients with precancerous lesions/early gastric adenocarcinoma than in healthy controls.	100/75%
Liang et al. (76)	↑miR-18a	Plasma	Diagnostic. Discriminates between GC patients and healthy groups; reduced in postoperative samples compared to in preoperative samples	76/73%
Yörüker et al. (77)	↑lncRNA H19	Plasma	Diagnostic. Elevated in GC patients; decreased significantly upon surgical removal of gastric tumors	87.2/38.1%
Emami et al. (78)	↑miR-21, ↑miR-222	Plasma	Diagnostic. Increased in GC patients compared with the control group	86.7/72.2%, 62.5/56.2%
Jiang et al. (79)	↑miR-551b-5p	Serum	Diagnostic. Differentiate GC patients from healthy controls	77.5/80%
Chen et al. (80)	↑miR-421	plasma	Diagnostic. Detection of precancerous lesions and early GC	96.67/95.56%
Shi et al. (64)	↑miR-1246	serum/exosomes	Diagnostic. Differentiate GC patients with TNM stage I from healthy controls	85.71/74%
Guo et al. (81)	↑lncRNA-GC1	serum/exosomes	Diagnostic. Detection of early-stage GC, especially for patients with GC with negative standard biomarkers	88.24/82.29%
Shao et al. (65)	↓miR-212	serum	Diagnostic /Prognostic. Poor prognosis predictor	95.1/78.7%

↑- *High-level*; ↓- *low-level*.

## Exosomes

Exosomes are small membrane vesicles of endocytic origin, with a diameter between 30 and 150 nm. They are secreted by cells into the environment in response to various physiological or pathological processes (82). The majority of the circulating transcriptome secreted by tumor cells is assisted by exosomes that protect cargos to travel along with the bloodstream. Through them, tumor cells can alter the microenvironment, influence the anti-tumor immune response, and modulate invasion and angiogenesis (83, 84). As a result, these regulatory properties of tumor cell-derived exosomes are essential in promoting tumor growth. Once released into the bloodstream or tumor microenvironment, the exosomes can interact with adjacent cells producing varied biological effects: direct exosome-cell stimulation or via transferred exosome cargo (85).

Due to their endosomal origin, they can be identified based on surface proteins such as CD81, CD62, and CD9 (86). In addition, they may have cell-specific or tumor-associated antigens on the surface reflecting the identity of the cells they come from (67, 87, 88).

This specific identity of the exomes makes them important candidates as reliable biomarkers. So, exosomes are macrovesicles rich in transcripts specific to tumors (89) that could be used as a biomarker of cancer diagnosis, progression, and metastasis.

It was shown that based on their composition GC exomes promote proliferation in an autocrine manner (90) or by activation of MAPK/ERK pathways (91) and PI3K/Akt (91). Exosomes are involved in mesothelial invasion and tumor dissemination within the abdominal wall and diaphragm (92), inducing apoptosis and mesothelial-to-mesenchymal transition (MMT), with mesothelial barrier destruction and peritoneal fibrosis (93) by enhancing the expression of fibronectin 1 and laminin gamma 1 (94).

GC-derived exosomes are involved in the modulation of tumor immunity. Thus, circulating exosomal PD-L1 was shown to be an independent prognostic factor in GC, associated with the immunosuppressive status of GC patients and decrease in CD4+ T cell count, CD8+ T-cell count, and granzyme B (95). Moreover, exosomal PD-L1 significantly decreased T-cell surface CD69 by increasing PD-1+ tumor-associated macrophages (TAMs), which by interacting with PD-L1+ cells can increase IL-10 production and CD8+ T cell dysfunction (96).

We can conclude that exosomal cargos are implicated in GC progression and modulation of tumor immunity via cellular communication and interactions in the tumor microenvironment.

## Conclusions

There is currently an abundance of work both in terms of clinical studies on different types of biomarkers and as a study methodology used to track these biomarkers in liquid biopsies. So much diversity in assays and principles of work has made liquid biopsy difficult to translate to the clinic, mostly due to the lack of reproducibility.

Before introducing liquid biopsies in current clinical use for surveillance, the methodologies should be standardized to ensure reproducibility, and proper controls should be developed and used. Quantification of CTCs, ctDNA, ncRNAs, cell heterogeneity analysis, and molecular modifications resulted from liquid biopsies assessment should be included in designing surveillance protocols based on international guidelines outlined from consistent evidence.

In this regard, an international consortium The International Liquid Biopsy Standardization Alliance (ILSA) was founded in December 2020 (97), with the role of promoting liquid biopsy. The organization aims to standardize work protocols, implement them and evaluate their performance and clinical utility.

However, several biomarkers obtained by liquid biopsy already proved their usefulness. Thus, CTC cut-off number (≥2), HER2 and PD-L1 expression on their surface showed to have clinical utility in therapeutic management, allowing a better selection of patients for specific therapies like trastuzumab or immune-checkpoints inhibitors. CtDNA offers the possibility to explore alterations in tumor DNA at the genetic and epigenetic level through assays such as CNVs, gene integrity, gene mutations, DNA methylation, and therefore proved to be essential for distinguishing tumor subtypes, and guide personalized therapy. Regarding ncRNA, a lot of studies highlighted their utility in early diagnosis and surveillance of cancer progression but it is difficult to select keys ncRNAs from a large number of candidates, however, their combination may shape-up the basis for the development of an early diagnostic or prognostic panel.

## Author Contributions

MC-E wrote the circulating tumor cells section and reviewed and edited the manuscript. LN wrote the circulating non-coding RNAs section. LM wrote the circulating tumor DNA section. CB wrote on the exosomes. DD wrote on the role of liquid biopsy in monitoring treatment response and CD wrote its role in surveillance for recurrent disease. All authors approved the submitted version.

## Funding

This work was supported by Grants of the Ministry of Research, Innovation and Digitization, CNCS/CCCDI – UEFISCDI, Project Number PN-III-P4-ID-PCCF-2016-0158 (contract PCCF17/2018), within PNCDI III and Project Number TE 36/2020 (PN-III-P1-1.1-TE-2019-1864) within PNCDI III.

## Conflict of Interest

The authors declare that the research was conducted in the absence of any commercial or financial relationships that could be construed as a potential conflict of interest.

## Publisher's Note

All claims expressed in this article are solely those of the authors and do not necessarily represent those of their affiliated organizations, or those of the publisher, the editors and the reviewers. Any product that may be evaluated in this article, or claim that may be made by its manufacturer, is not guaranteed or endorsed by the publisher.
